# Understanding the Concept of Pre-Clinical Autoimmunity: Prediction and Prevention of Systemic Lupus Erythematosus: Identifying Risk Factors and Developing Strategies Against Disease Development

**DOI:** 10.3389/fimmu.2022.890522

**Published:** 2022-06-03

**Authors:** May Y. Choi, Karen H. Costenbader

**Affiliations:** ^1^ Brigham and Women’s Hospital and Harvard Medical School, Boston, MA, United States; ^2^ Department of Medicine, University of Calgary, Calgary, AB, Canada; ^3^ Cumming School of Medicine, McCaig Institute for Bone and Joint Health, Calgary, AB, Canada

**Keywords:** systemic lupus erythematosus, prevention, biomarkers, risk factors, pathogenesis

## Abstract

There is growing evidence that preceding the diagnosis or classification of systemic lupus erythematosus (SLE), patients undergo a preclinical phase of disease where markers of inflammation and autoimmunity are already present. Not surprisingly then, even though SLE management has improved over the years, many patients will already have irreversible disease-related organ damage by time they have been diagnosed with SLE. By gaining a greater understanding of the pathogenesis of preclinical SLE, we can potentially identify patients earlier in the disease course who are at-risk of transitioning to full-blown SLE and implement preventative strategies. In this review, we discuss the current state of knowledge of SLE preclinical pathogenesis and propose a screening and preventative strategy that involves the use of promising biomarkers of early disease, modification of lifestyle and environmental risk factors, and initiation of preventative therapies, as examined in other autoimmune diseases such as rheumatoid arthritis and type 1 diabetes.

## 1 Introduction: Prediction and Possibly Prevention of SLE in the Near Future

Systemic lupus erythematosus (SLE) is a chronic autoimmune disease characterized by immune dysregulation and systemic inflammation, leading to progressive and irreversible multi-organ damage. Although SLE is relatively uncommon [SLE affects ~25 to 50 per 100,000 persons in the United States (1, 2)], it disproportionately affects young women during their prime reproductive years, particularly those of non-White ancestry (3, 4). SLE remains among the leading causes of mortality in young females, underscoring its impact as an important public health issue (5, 6). With the discovery of more risk factors for SLE including genetics and environmental/lifestyle risk factors our ability to estimate SLE risk is improving, and thus so is the identification of patients who are at high versus low risk of this complex autoimmune disease.

A better understanding of SLE pathogenesis may enable earlier and more accurate identification of at-risk patients, as well as the discovery of therapeutic targets, and the design of prevention trials. However, since the breakthrough and serendipitous discovery of the Lupus Erythematous (LE) cell and its role in SLE pathogenesis in 1948 (7), are we any closer to achieving this goal? The LE cell provided evidence that autoantibodies are a key player in SLE pathogenesis, which are generated by a dysregulated immune system leading to immune complex formation and deposition, and subsequent inflammation and organ damage. In the 75 years that followed the LE cell identification, there was an explosion of serologic tests and technologies developed to detect autoantibodies, most centrally the antinuclear antibody (ANA) test, to aid in the diagnosis or classification of SLE [reviewed in (8)].

SLE is notoriously difficult to diagnose and classify because of the heterogeneity and non-specificity of clinical signs and symptoms in early disease. The diagnosis of SLE is thus frequently delayed such that by the time a formal diagnosis is confirmed, irreversible organ damage has already occurred. There are reports that the diagnosis of SLE is delayed by a median of 47 months, with patients submitting to an average of 10 consultations and evaluation by three different physicians before a diagnosis is finally made (9). A delay in SLE diagnosis has been associated with worse outcomes including higher disease activity, organ damage, lower quality of life, and remarkably increased healthcare costs (9). Organ damage occurring early in the disease course also has a negative impact on SLE patients, as it is associated with further damage, development of comorbidities and early mortality (10, 11). The classification criteria for SLE have been through several iterations to improve sensitivity and specificity, with the most recent criteria being the American College of Rheumatology (ACR)/EULAR (European League Against Rheumatism) 2019 criteria (12, 13). Unlike the others, one of the major differences with the new criteria is that it uses the “ANA at a titer of ≥1:80 on HEp-2 cells or an equivalent positive test at least once” as an entry criterion.

Despite advances in therapy, such as the recent approval of several new drugs (anifrolumab, voclosporin, and a new indication for belimumab) (14–16), without timely and accurate diagnosis to allow the initiation of evidence-based therapy, patients with SLE will continue to be at increased risk for morbidity, disability, and premature death secondary to cardiovascular events (e.g., strokes and myocardial infarction), malignancy, and infection, driven by uncontrolled inflammation (6, 17). Furthermore, antimalarials continue to be the mainstay therapy in SLE. Hydroxychloroquine (HCQ) has been shown to reduce SLE flares (lupus nephritis in particular), organ damage, pregnancy complications, cardiovascular events and survival (18–23). There is also evidence to suggest it can delay the onset of SLE, prompting a clinical trial that is currently underway to answer whether it can be used as a preventative therapy (18).

Emerging research suggests that our increasing knowledge about risk factors and biomarkers for SLE could lead to the identification of those at highest risk, and potentially then to early interventions *prior to the onset of symptoms*, to intercept and prevent this often-devastating disease. We review how current understanding of the development of SLE is contributing to progress in the identification of those who are developing disease, and how genetic and population risk factor studies are leading to the potential for disease prevention through early identification, environmental or lifestyle changes, and therapeutic interventions.

## 2 The pathogenesis of pre-clinical SLE and important biomarkers and risk factors

**Figure 1:**
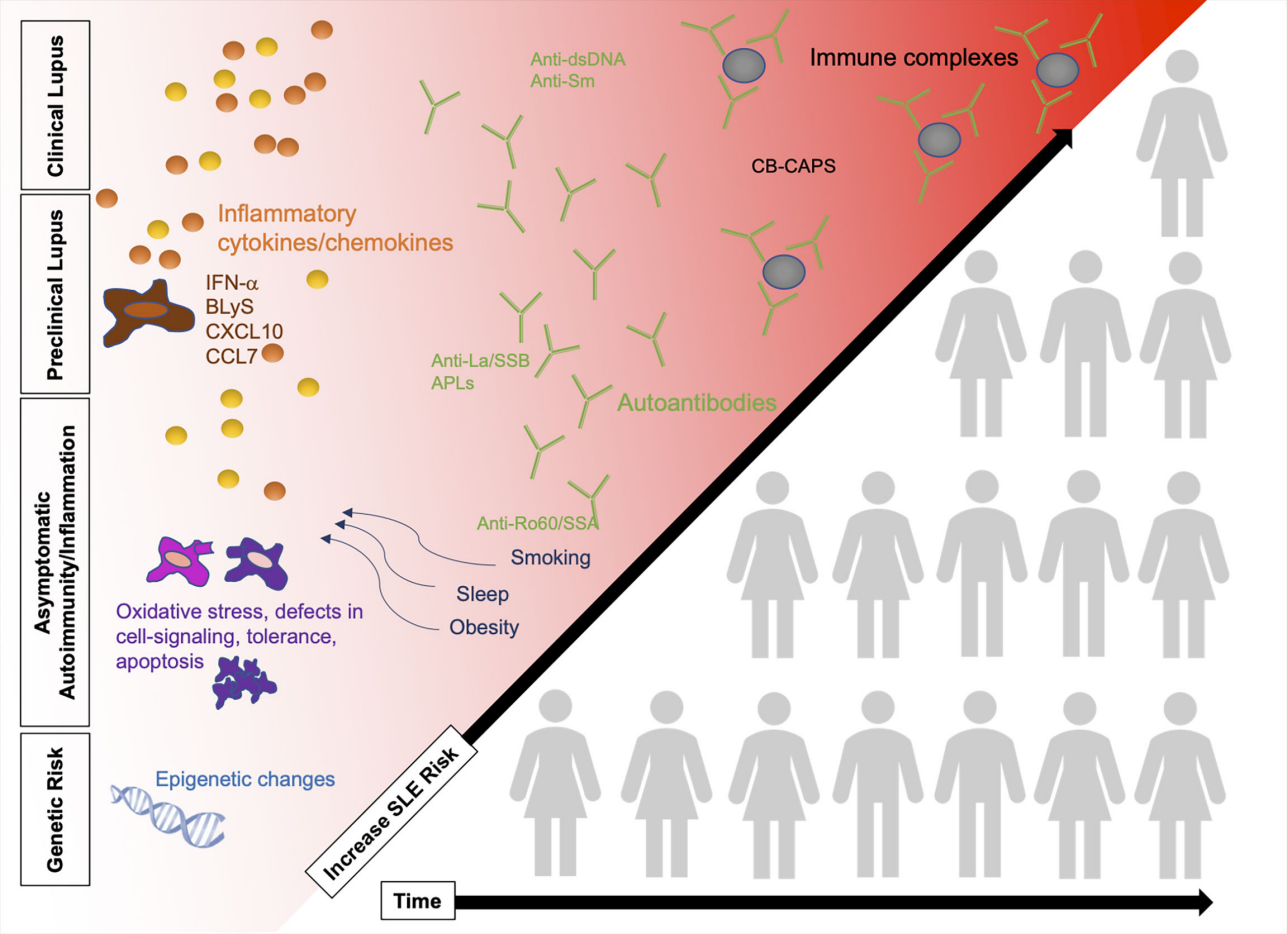
SLE pathogenesis in four phases, increasing in SLE risk over time as patients accumulate risk factors. Changes in the immune system are detected prior to the diagnosis of clinical SLE including presence of autoantibodies, cytokines, and immune complex deposition. Some patients (illustration not representative of actual pre-clinical/clinical SLE population) will progress over time to clinical SLE while others remain in the earlier stages of preclinical SLE. Refer to **Figure 2** for potential points for early risk assessment and intervention opportunities. BLyS, B-cell lymphocyte stimulator; IFN, interferon; SLE, systemic lupus erythematosus.

Understanding of the etiopathogenesis of SLE is evolving [reviewed in (24)]. The currently accepted model for multiple complex autoimmune diseases is that development takes place over time prior to diagnosis and in several stages (**Figure 1**). This next section will review the three phases that precede the diagnosis of SLE: 1) genetic risk, 2) asymptomatic autoimmunity and inflammation, and 3) early symptoms of lupus. As we discuss each phase, we will describe potential avenues of disease prevention including biomarkers for early disease detection and modifiable risk factors.

### 2.1 Genetic Risk

SLE likely begins and is accelerated by a complex interplay between genetic risk, lifestyle and environmental risk factors and immune dysregulation. When individuals who possess SLE genetic risk alleles are exposed to environmental risk factors throughout their lives, synergistic interactions may take place, accelerating the onset of autoimmunity and inflammation. About 5-12% of subjects with a first-degree relative with SLE will develop the disease in their lifetime, whereas in persons with a congenital deficiency of the complement component C4, this risk can increase to 90% (25). Children who develop SLE appear to have a larger contribution of known SLE genetic risk, in particular non-HLA genes, than do adults with SLE, and thus the contribution of environmental exposures to SLE susceptibility may be increasingly important with advancing age (26, 27).

A series of landmark genome-wide association studies (GWAS) over the past decade in SLE have greatly expanded our understanding of the genetic basis of SLE [reviewed in (28, 29)]. To date, over 100 SLE susceptibility loci have been identified, predominantly in European and Asian populations, explaining up to 30% of SLE heritability (30–44). These include alleles in the Major Histocompatibility Complex (MHC) region (multiple genes), some of the Fcγ receptors, *ATG5, BLK, BANK1, IRF5 (interferon regulatory factor 5), ITGAM, PDCD1, PTPN22, PXK, SPP1, STAT4, TNFSF4, TNFAIP3*, *XKR6*, and deficiencies in complement components (29). Many of these genes belong to important pathways involved in immune complex clearance, host immune signal transduction, and pathways involving interferon (IFN), a key driving cytokine in many cases of SLE.

SLE-associated genes involved in the innate immune system have been gaining interest because of the “IFN signature”. Patients with SLE and high levels of IFN-α tend to have more severe disease manifestations (45). Normally, type I IFNs are produced during early response to viral infections and promote dendritic maturation and proinflammatory cytokines. This has several important effects on the immune system including the stimulation of the Th1 pathways, promotion of B-cell activation for autoantibody production, and regulation of apoptosis. One of these genes is *IRF5*, which regulates type I IFN-responsive genes. Outside of the MHC, it is one of the most strongly and consistently SLE-associated with a modest contribution to SLE risk (odds ratio 1.5) (46). The rs7574865 SNP risk variant of *STAT4* has also shown to confer increased sensitivity to IFN-α signaling in peripheral blood mononuclear cells of SLE patients, and is associated with more severe disease, early disease onset and production of antibodies to double-stranded DNA (dsDNA) (47). Together, *IRF5* and *STAT4* have an additive effect for increased risk of SLE development (48). Additional genes that influence the IFN pathway and innate immune signaling include *IRAK1*, which is found on the X chromosome and therefore is thought to contribute increased SLE risk among females (49), and osteopontin, which is also associated with early disease onset (50), as well as *IRF7, IFIH1*, and *TYK2*.

Other risk factors for SLE development are genes linked to the MHC, primarily *HLA-DRB1* in the MHC class II region (51). HLA molecules play a key role in autoantibody production as demonstrated by one Japanese study that identified both SLE risk signature and autoantibodies to ribonucleoprotein (RNP), SSA/Ro60, SSB/La, cardiolipin were localized to the peptide binding groove of *HLA-DRB1* and anti-Sm to *HLA-DPB1* (52). Multiple genes involved in the adaptive immune response and autoantibody production have also been linked to SLE risk such as *PTPN22* (53) and *BANK1* with three functional variants that lead to an altered B cell activation threshold to increase SLE risk (41).

Given that SLE is multifactorial and multigenic, an individual’s risk for SLE development cannot be well estimated using only known genetic risk factors. Several similar weighted genetic risk scores (GRS) have been developed to try to estimate an individual’s cumulative genetic susceptibility to SLE risk (54). A high GRS has been associated with earlier onset SLE and more severe disease phenotypes (55). Overall, men with SLE also appear to have slightly higher GRS than do women with SLE, suggesting that there is a stronger genetic component of disease among families with male SLE patients and perhaps that environmental or hormonal factors contribute to lowering the threshold for the development of SLE more among females than males (or, conversely, environmental, or hormonal factors may raise this threshold in males) (56). Other studies have also demonstrated greater SLE risk when genetic and environmental interactions are combined such as vitamin D status in those with *CYP24A1* alleles (57), current/recent smoking and GRS (54).

Future genetic studies will likely reveal increased numbers of genetic biomarkers, further refining our understanding of SLE risk and pathogenesis. Large genetic studies in more diverse racial and ethnic groups are still necessary, as most SLE GWAS to date have studied subjects of European or Asian ancestry. Research and development of models that incorporate environmental risk factors will hopefully hone our ability to identify those who are at high risk of developing SLE, and lead to new therapeutic targets.

### 2.2 Asymptomatic Autoimmunity and Inflammation

Some individuals genetically susceptible to SLE will transition into a period of asymptomatic autoimmunity and inflammation prior to the development of overt clinical manifestations. Which individuals will progress and why? These are key questions that we are still trying to answer. Thus far, studies have pointed to environmental risk factors, some known and others yet to be discovered, as potential triggers for this transition. These events likely act by both separate and overlapping biological pathways, including but not limited to increasing oxidative stress, loss of immune tolerance, autoantibody formation, complement activation and immune complex deposition, epigenetic modifications, and upregulation in cytokine expression (58). In this pre-symptomatic phase where there is already evidence of early immune changes, can we use this our advantage to identify these at-risk patients earlier? And if we could identify the earliest changes of SLE, could we “turn it off” or move a person “backwards” on their trajectory towards SLE? In this next section, we will highlight important biomarkers and potential interventions as we review the different pathways of autoimmunity and inflammation in SLE pathogenesis.

#### 2.2.1 Increased Oxidative Stress

Oxidative stress, which is defined by an imbalance between the production and neutralization of reactive oxygen intermediates (ROI), is normally utilized by phagocytic cells to eliminate pathogenic organisms. However, in SLE, this is increased leading to abnormal activation and processing of cell-death signals and autoantibody production [reviewed in (59)]. Endogenous sources of oxidative stress include increased ROI production in mitochondria, NADPH oxidase enzymes in phagocytes, endothelial cells, T cells, and B cells (60, 61). Ultra-violet (UV) radiation, viral and bacterial infections, and chemical exposure have been implicated to be environmental sources of oxidative stress. Oxidative stress not only induces T-cell dysfunction and propagation of oxidative modification of self-antigens leading to systemic inflammation, but it also damages various organ systems resulting in renal, cardiovascular, and cutaneous disease/comorbidities in SLE (62–64).

Currently, there are no biomarkers of oxidative stress in routine clinical use. Potential biomarkers that have been correlated with disease activity in established SLE patients include increased modification of serum albumin (65), urinary levels of F2 isoprostane (66), and serum nitric oxide levels (67). Future studies are still needed to determine if these biomarkers and others can help diagnose pre-symptomatic disease. Potential antioxidant therapies for SLE include N-acetylcysteine and rapamycin, but their role in preclinical disease is unclear (68, 69). On the other hand, dietary intake of antioxidant vitamins (vitamins A, C, and E and α-carotene, β-carotene, cryptoxanthin, lycopene, lutein, or zeaxanthin) has not been found to decrease SLE risk in epidemiologic studies (70, 71).

#### 2.2.2 Break in Immunological Tolerance

Loss of self-tolerance occurs in SLE when autoantibodies target nuclear self-antigens that are released into the extracellular space and exposed to the immune system [reviewed in (72)]. Abnormalities in apoptosis, NETosis, and histone modifications are thought to be involved in this process. Apoptosis is an important source of autoantigens in SLE and it has been shown that many of the nuclear autoantigens (e.g., DNA, Ro, La, and small nuclear RNP) that are targeted in SLE are clustered in blebs at the surface of apoptotic cells where oxidative modification can occur (63, 73). NETosis is a specialized form of neutrophil cell death that has also been implicated as another potential source of autoantigens (74). During NETosis, structures termed neutrophil extracellular traps (NETs) are extruded by neutrophils to entrap and dismantle bacteria, viruses, fungi, and parasites. These NETs include fibrillary networks of DNA, citrullinated histones, and granule peptides such as cathepsin G, neutrophil elastase, and myeloperoxidase. In SLE, apoptosis and NETosis are increased, resulting in an excess load of nuclear autoantibodies (72, 74).

However, these on their own are unlikely to break immunological tolerance as several studies were not able to induce immune activation by immunizing mice with apoptotic cells/blebs or NETs (75, 76). A deficiency in clearance of apoptotic cells and/or NETs due to intrinsic phagocyte defects and absent/deficient serum factors are thought to lead to an enduring exposure of modified proteins such as histones in the immune system (77). These modified proteins are regarded as neoantigens that are no longer perceived as endogenous and subsequently elicit an autoimmune response. It can also stimulate an inflammatory response through the activation of nucleic acid recognition receptors (e.g., members of the Toll-like receptor (TLR) family), which are important in viral and bacterial defense and associated with type I IFN production (discussed in *2.2.4 Cytokines/Chemokines*). Improving the clearance of apoptotic cells and/or NETs may therefore be potential therapeutic targets for SLE or SLE prevention.

#### 2.2.3 Autoantibodies

In addition to apoptotic cells and NETs, other important sources of autoantigens include neoantigens generated from necrotic cells under the influence of processes like oxidation and cleavage and infectious agents (e.g., single-stranded RNA, double-stranded RNA, and DNA). Autoantibodies and cytokines are produced by B lymphocytes that process and present these antigens. Autoantibodies can form immune complexes with their antigen, which can lead to organ damage through immune complex deposition and local and systemic inflammation. In a positive feedback loop, autoantibodies can then induce NETosis, and immune complexes can stimulate plasmacytoid dendritic cells to produce pro-inflammatory cytokines including IFN-α which can incite further NETosis. In SLE, intrinsic abnormalities of B-cell and T-cell interaction also contributes to the production of autoantibodies [reviewed in (78)]. In SLE, these cells are hyperresponsive to stimuli resulting in the production of higher quantities of autoantibodies and cytokines. Furthermore, defects in immune tolerance permit the survival of dangerous autoreactive B cells that lead to further production and diversification of harmful autoantibodies in a process called epitope spreading (79, 80). Early in the disease course, an antibody response might begin with a particular epitope, and this is then later followed by a spread of the response to other epitopes in the same polypeptide (intramolecular) and in other distinct but structural similar molecules (intermolecular) (81). In **Table 1**, we summarize common SLE autoantibodies, their clinical associations, and onset prior to the diagnosis of SLE (82–85).

**Table 1:** SLE autoantibodies, clinical significance, and time to SLE onset.

Antibody Target		SLE Clinical Significance	Time to SLE Onset^1^
SSA/Ro60		Subacute cutaneous SLE Lymphopenia Neonatal lupus In pediatric SLE, milder disease (cutaneous, musculoskeletal) Protective with SSB/La (less renal and neurologic disease)	Up to 8.1-9.4 years (mean 2.3-2.97 years)
SSB/La		Subacute cutaneous SLE Neonatal lupus Leukopenia Serositis Protective with SSA/Ro60 (less renal and neurologic disease)	Up to 7.0-8.1 years (mean 0.6-2.83 years)
Cardiolipin		Part of classification criteria Antiphospholipid syndrome Pulmonary hypertension Decreased survival	Up to 7.6 years (mean 2.29 years)
dsDNA		Part of classification criteria Lupus nephritis Disease activity Pathogenic	Up to 6.6-9.3 years (mean 1.24-2.0 years)
U1-RNP		Leukopenia Neuropsychiatric SLE Raynaud’s Musculoskeletal involvement Lung involvement	Up to 7.2-7.5 years (mean 0.20-1.2 years)
Histone		Drug-induced SLE Neuropsychiatric SLE Pathogenic	Up to 6.5 years (mean 1.9 years)
Sm (U2-U6 RNP)		Part of classification criteria Serositis Lupus nephritis Neuropsychiatric SLE	Up to 1.1-8.1 years (mean 0.47 years)
Ro52/TRIM21		Hematologic involvement with SSA/Ro60 Neonatal lupus More severe disease (renal)	Predictive of progression to SLE in patients followed over two years
C1q		Lupus nephritis Hypocomplementemic urticarial vasculitis with or without SLE	Detected in incomplete SLE patients but infrequently, timing unknown
β2GP1		Part of classification criteria Antiphospholipid syndrome Pathogenic	Unknown
β2GP1 domain 1		Antiphospholipid syndrome	Unknown
High Mobility Group Proteins		Disease activity	Unknown
Ku		Raynaud’s Myositis Arthritis	Unknown
Nucleosomes and Chromatin		Lupus nephritis with more severe renal failure Disease activity Pathogenic	Unknown
PCNA		Lupus nephritis Neuropsychiatric SLE Thrombocytopenia	Unknown
PS/PT		Antiphospholipid syndrome	Unknown
Ribosomal P		Lupus nephritis Neuropsychiatric SLE Lupus hepatitis Disease activity	Unknown

1. Based on Arbuckle et al. (83), Eriksson et al. (84), Munoz-Grajales et al. (85), and Olsen et al. (86).

2. β2GP1, beta 2 glycoprotein 1; dsDNA, double-stranded DNA; PCNA, proliferating cell nuclear antigen; PS/PT, Phosphatidylserine/Prothrombin; RNP, ribonucleoprotein; SLE, systemic lupus erythematosus; TRIM21, Tripartite motif containing-21.

SLE is thus a paradigmatic autoimmune disease, with formation and detection of a wide range of autoantibodies, some of which are more SLE-specific and more pathologic than others. Autoantibody detection has long been a valuable and effective approach to the diagnosis, classification and prognostication with a wide range of established systemic autoimmune rheumatic diseases (SARD), including SLE (87). However, the exact contribution of autoantibody testing to the identification of subclinical and very early SLE is still to be determined. In a seminal study by Arbuckle et al. (83), a serum biobank and database established by the American military was queried and SLE-related autoantibodies were found in stored blood up to 9.4 years (mean 3.3 years) before the onset of SLE symptoms and eventual diagnosis. Other studies have confirmed similar findings (84, 88–92). Anti-SSA/Ro60 antibodies typically appeared first (83, 91, 92). Anti-SSB/La and anti-phospholipid antibodies have been reported to appear next (83). IgG and/or IgM anti-cardiolipin antibodies were detected in 18.5% of patients with mean onset of 3.0 years prior to the diagnosis of SLE and up to 7.6 years before SLE diagnosis (93). Anti-dsDNA anti-Sm, and anti-RNP antibodies (mean 3.4 vs. 1.2 years; p=0.005) appear later (83, 91, 92). Other studies have also demonstrated that anti-dsDNA and anti-Sm antibodies in non-SLE or early undifferentiated connective tissue disease patients are predictive of SLE evolution (88, 94, 95). A positive ANA test, a test used to screen for the presence of autoantibodies, has been reported to appear up to 9.2 years (mean 2.25 years) prior to SLE diagnosis or classification. As SLE progressed before and after diagnosis or classification, new autoantibodies steadily accumulated, consistent with other literature supporting increased epitope spread over time (85, 92, 96, 97).

The *absence* of specific autoantibodies in SLE or the presence of others may also help to identify those who are at *lower risk* of progression to SLE. ANAs are non-specific and found in up to 20% of healthy subjects, and are more common in females, with increasing age, and in the setting of infection, lung, and autoimmune thyroid disease (98–100). Anti-dense fine speckled 70 (DFS70) antibodies may be a useful biomarker to *rule out* the diagnosis of SLE as they are rarely found in SLE patients. In an international study of 1137 patients with SLE followed from inception in the Systemic Lupus International Collaborating Clinics (SLICC) cohort, only 1.1% had monospecific (no other detectable autoantibodies) anti-DFS70 antibodies (101). Thus, the presence of anti-DFS70 antibodies may help to discriminate between those who are ANA-positive healthy subjects versus those with SLE. Anti-C1q autoantibodies, which are associated with lupus nephritis (102), were infrequently found in patients with incomplete SLE in a small cross-sectional study of 70 patients (86). The authors suggest that although it remains undetermined whether this autoantibody could be a predictor of SLE risk, the presence of an elevated anti-C1q antibody in a patient with incomplete SLE might raise concerns for SLE or more specifically, lupus nephritis (86).

One of the challenges of identifying novel predictive autoantibodies for SLE development is that although over 200 different autoantibodies have been described in SLE, only 10% have been made widely available as diagnostic assays approved by regulatory authorities; most are still for research purposes only (10). Furthermore, most studies of these novel autoantibodies in SLE have been small and cross-sectional in design, without consideration of hallmarks of early disease or variable longitudinal disease course and outcomes, even though autoantibody test results may vary over time. The parameters associated with this longitudinal variation, such as the impact of medical therapies on antibody responses, also have not been well studied.

There has been a call for future exploration of novel autoantibody biomarkers given the non-specificity of ANA for SLE (11, 12). Investigators at the University of Toronto examined approximately 200 ANA-positive patients without established SARD, using a custom antigen microarray of 144 established and novel autoantibodies (85). They found that the majority of patients who tested negative for most current commercially available autoantibodies were positive for autoantibodies on their custom microarray. Anti-Ro52/Tripartite motif containing-21 (TRIM21) autoantibodies were predictive of SARD progression over the next two years (defined by the 1997 ACR criteria for SLE (103), 2013 ACR-EULAR criteria for systemic sclerosis (104) or 2016 ACR–EULAR criteria for Sjögren’s syndrome (105)), with positive predictive value of 46% and negative predictive value of 89%. To close the ‘seronegative gap’, more studies of novel disease-specific autoantibody biomarkers are needed and will help to identify valid predictors of disease evolution, potentially enabling identification and treatment of patients with SLE in these early stages (10).

#### 2.2.4 Cytokines/Chemokines

Increased IFN-α activity is an important contributor to SLE pathogenesis because of its involvement in the induction of B-lymphocyte stimulator (BLyS) and DNA- and RNA- protein binding autoantibody specificities. BLyS plays a key role in regulating B cell survival and differentiation, which is central to autoantibody production and class switching. Drugs blocking BLyS activity (belimumab), and more recently, the type I IFN receptor subunit 1 (anifrolumab), have reduced disease activity in patients with SLE in large clinical trials and are now approved therapies for SLE treatment (14, 16).

In a case-control study by Munroe et al. of SLE patients and matched healthy controls, serum collected prior to and at/after SLE classification were analyzed (92). Prior to SLE classification (average timespan of 4.3 years), upregulation of IFN-associated mediators, as observed with autoantibodies, accumulated over a period of years, and then plateaued close to the time of disease classification (p<0.001). The most important predictor of increased IFN-α activity was the number of positive autoantibodies (p<0.001). Increased circulating IFN-α activity and BLyS levels were also detected shortly before subjects met SLE classification criteria (p≤0.005), suggesting that this may be a turning point in SLE pathogenesis where immune dysregulation is amplified by positive feed-forward mechanisms. Other studies have also showed that early SLE patients have exacerbated type I IFN signatures, their autoantibodies specificities have already class-switched to IgG isotypes (106), and autoantibody containing immune complexes drive type I IFN activation (107–110).

Although IFN-α activity may be an important contributor to SLE progression, not all SLE patients (only ~25%) have increased IFN-α activity preceding SLE diagnosis or classification (92). Hence, other forms of immune dysregulation likely accompany IFN-α activity, such as type II IFN (IFN-γ). IFN-γ is important in mediating the crosstalk between innate cells and lymphocytes, breaking self-tolerance and enabling the activation and persistence of autoreactive B cells (111). It modulates TLR regulation to facilitate autoantibody production, antigen presentation, and recruitment of lymphocytes to germinal centers (111). It can also drive the production of IFN-α and BLyS levels, leading to inflammation, B cell activation and autoantibody production. Munroe et al. further found increased levels of circulating IFN-γ in pre-clinical SLE patients prior to detectable upregulation of IFN-α and autoantibody positivity, as well as dysregulation of the chemokines IP-10 (CXCL10) and MCP-3 (CCL7) (92). Other mediators that have been implicated in SLE pathogenesis and are elevated years before SLE classification include IL (interleukin)-12p70, MIG, IL-4, IL-5, and IL-6 (91). These chemokines, which aid in the recruitment of cells to sites of inflammation, may also be important biomarkers in early pathogenesis of SLE.

#### 2.2.5 Complement Activation

Complement activation is responsible for much of the systemic inflammation and tissue damage in SLE [reviewed (112)]. All three pathways of complement activation are involved in SLE, with the classical pathway, activated by antigen-antibody complexes, being the most important in SLE pathogenesis. Low complement C3, C4 and CH50, levels are diagnostic and disease activity biomarkers in SLE (113). However, they are not always reliable as they are influenced by the acute phase response, individual differences in complement gene copy number and expression, and variability in protein catabolism and synthesis (114).

To overcome the limitations of measuring C3 and C4, assays to measure cell-bound activation (split) products (CB-CAPS), such as erythrocyte-bound C4d (EC4d) and B lymphocyte-bound C4d (BC4d), have recently been developed. These are formed upon activation of the complement cascade and reflect complement activation rather than the levels of the individual protein. These are measured using EDTA anti-coagulated blood by flow cytometry which can be labor intensive, but on the other hand, sample processing is usually minimal, no centrifugation is needed, and it does not require low temperature for storage and transportation.

CB-CAPS are promising SLE biomarkers, shown to be more sensitive than C3, C4, and anti-dsDNA for the SLE diagnosis (115, 116), and more prevalent in patients with probable SLE. When used in combination with a proprietary panel of other autoantibodies, one study reported these biomarkers were able to identify patients with a greater than three-fold increased risk of developing SLE and were slightly better than complements or anti-dsDNA alone at predicting transition to SLE among patients with undifferentiated connective tissue disease [reviewed in (117, 118)]. These results suggest that complement activation may also occur early in the evolution of SLE and be an important feature in patients with suspected SLE.

#### 2.2.6 Lifestyle and Environmental Risk Factors Related to SLE Risk (With a Focus on Those That Are Potentially Modifiable)

The number of factors beyond age, race, sex, family history, and genetics that are strongly associated with risk of developing SLE has been growing in recent years. Multiple large cohort studies have contributed to our understanding of how lifestyle, behavioral, psychosocial, and environmental risk factors may converge and synergize with underlying genetic risk. This likely leads to an acceleration of underlying and brewing autoimmunity, allowing it to manifest in SLE. These factors include current cigarette smoking, obesity (in particular, at younger ages), childhood and adult trauma, stress, post-traumatic stress disorder, low or no alcohol intake, environmental air pollution, environmental silica, and hormonal exposures and reproductive factors among women [reviewed in (58, 119)]. While is it not known whether these environmental risk factors work *via* similar or disparate biologic pathways, nor whether they are perhaps also inextricably linked to other societal risk factors that are more difficult to measure, the picture of how and the extent to which they contribute to SLE susceptibility is coming into focus. Gene-environment interactions likely contribute to SLE risk, and only a handful of these specific interactions have been discovered to date (54, 57).

In a recent, large, prospective evaluation of healthy lifestyle behaviors and SLE risk using the Nurses’ Health Study (NHS) and NHSII, adherence to multiple healthy behaviors (healthy diet (highest 40th percentile of the Alternative Healthy Eating Index), regular exercise (performing at least 19 metabolic equivalent hours of exercise per week), never smoker or past smoker, moderate alcohol consumption [drinking ≥5 gm/day alcohol), and maintaining a healthy body weight (body mass index <25 kg/m^2^)] was associated with a lower risk of SLE development overall (120). There was a 19% reduction for each additional healthy behavior and an even greater reduction (22%) was observed for the risk of dsDNA positive SLE. Strikingly, the risk of SLE was *half as high* among those with the best adherence to healthy lifestyle behaviors compared to among those with the poorest adherence. Overall, the population attributable risk, or the proportion of the risk in this population that could be attributed to these five modifiable lifestyle risk factors was 47.7% [95% confidence interval (CI) 23.1-66.6%]. These results suggest that lifestyle behaviors likely work synergistically to influence the risk of SLE and potentially produce stronger effects together than individually *via* common biological pathways including production of autoantibodies and dysregulation of pro-inflammatory cytokines. Moreover, although much work remains to be done in disentangling the specific pathways by which these environmental risk factors may be related to SLE pathogenesis, this also suggest that much of SLE may be preventable with lifestyle change, a somewhat revolutionary concept.

Many potential biologic mechanisms and synergies are possible. For example, exposure to obesity and toxic components of cigarette smoke both cause oxidative stress (121). This, in turn, increases intracellular levels of reactive oxygen species to damage DNA forming immunogenic DNA adducts, thereby promoting dsDNA antibody production (**section 2.2.3**) (122–124). In the NHS and NHSII cohorts, cigarette smoking was associated with a higher risk of anti-dsDNA positive SLE than never smokers [hazard ratio 1.86 (95%CI 1.14-13.04)] (125), a finding confirmed in other studies (126, 127). In addition to causing oxidative stress (*section 2.2.1*), the by-products of smoking could also augment autoreactive B cells in the native repertoire (126) and induce pulmonary ANA in the lungs of exposed mice (128). Alcohol consumption, on the other hand, contains several compounds such as ethanol and antioxidants, that can potentially counteract the changes induced by smoking and obesity including inhibiting key enzymes in DNA synthesis (129, 130). Moderate alcohol intake (≥5 gm/day or >0.5 drinks/day) was associated with a decreased risk of incident SLE in NHS and other studies [hazard ratio 0.61 (95%CI 0.41-0.89)] (131).

Although the association between SLE risk and various diets is less clear in humans (132–134), murine models have demonstrated that low dietary fiber intake and Western-type diet (i.e., high in sugar, fat, refined grains, and red meat) were associated with increased autoantibody production in SLE-prone mice (135, 136). A murine study also demonstrated that in mice genetically susceptible to SLE, sleep deprivation was associated with an earlier onset of disease and accelerated production of autoantibodies (137). Among women followed in the Black Women’s Health study, a diet high in carbohydrates was associated with increased risk of developing SLE (132). The association between lack of sleep (less than the recommended 7 hours a night) and SLE risk in humans has been reported in several studies (138, 139). In a prospective study of 436 non-SLE relatives of SLE patients, relatives were more likely to transition to SLE if they reported sleeping less than seven hours a day [odds ratio 2.8 (95%CI 1.6-5.1)] (138).

Many lifestyle factors associated with SLE development increase levels of pro-inflammatory cytokines (*section 2.2.4*). Smoking increases BLyS expression (128), Tumor necrosis factor alpha (TNF-α), and IL-6 (140, 141). Among positive ANA women, elevated BLyS and lower IL-10 (an anti-inflammatory cytokine) levels could be found among current smokers (142). Both TNF-α and IL-6 also play important roles in the modulation of insulin resistance (121). Adipose tissue, in particular visceral fat, secretes pro-inflammatory adipocyte-derived cytokines and exhibit higher levels of C-reactive protein (CRP), TNF-α receptor 2, and IL-6 than non-obese individuals (143). Alcohol, on the other hand, suppresses TNF-α, IL-6, IL-8, and IFN-γ to counteract systemic inflammation (129, 130). In sleep-deprived individuals, increased levels of IL-6, TNF-α have been observed in addition to its role in impairing the function of T cells and CD4 regulatory T cells, which are important in self-tolerance (**section 2.2.2**) (144–148). Sleep disturbances in individuals who have had childhood or adult trauma, post-traumatic stress disorder or occupational stress from working nightshifts or rotating shifts, may also explain why these factors have also been linked to SLE onset (149–155). Systemic inflammation with elevated TNF, IL-6 and CRP levels is also found in these conditions (150, 156–164).

Other environmental and occupational related risk factors, including chemical and physical exposures, have also been linked to SLE onset and mechanisms involving stimulation of cellular necrosis and relate to intracellular antigens with resulting inflammation and IFN upregulation. These exposures include crystalline silica dust (165–168), air pollution and other respiratory particulates (169, 170), heavy metals such as mercury (149), and agricultural pesticides (149, 171, 172). UV radiation is also thought to trigger SLE onset, and it has been shown in SLE patients and lupus-prone mice, that there is a rise in type I IFN signaling and expansion and prolonged activation of T cells following UVB exposure (173–175). The association of UV radiation and SLE risk however is likely complicated by its role in vitamin D3 synthesis in the skin, which has been hypothesized to *reduce* SLE risk (176). A more detailed discussion about vitamin D and its role in preventing SLE is found in **section 3.**

Use of exogenous hormones, oral contraceptive pills, and hormone replacement therapy have been associated with risk of SLE (177–179). Among recent oral contraceptive pill users, a dose response between oral contraceptive pill dose of ethinyl estradiol and SLE risk has been demonstrated (178). Estrogen is thought to induce autoreactivity by upregulating several genes involved in B cell activation and survival (*cd22*, *shp-1*, *bcl-2*, and *vcam-1*) and preventing B cell receptor-mediated apoptosis (180).

The association between infection and SLE is the Epstein-Barr virus (EBV) has been of interest for many years. The data on whether prior EBV infection is a risk factor for SLE development are still unclear [reviewed in (181)]. The release of EBV-encoded small RNA from infected cells is thought to induce type 1 interferon and proinflammatory cytokines *via* activating TLR-3 signaling (182). Another potential mechanism is through molecular mimicry between EBV and SLE antigens and epitope spreading. In a systematic review and meta-analysis of 25 case-control studies, a higher seroprevalence of anti-viral capsid antigen IgG [odds ratio 2.08 (95%CI 1.15-3.76)] and anti-early antigen antibody, a marker of viral replication, was observed in patients with *existing* SLE compared to health or nonhealthy controls [odds ratio 4.5 (95%CI 3.00-11.06)] (183). However, the results should be interpreted with caution given there was publication bias regarding recruitment, matching and reporting of blinded laboratory analysis and these studies do not address whether EBV is causally related to SLE. On the other hand, in a Danish population-based study, it was the EBV-serologic *negative* individuals that had an increased risk for SLE, particularly one to four years after serologic testing [standardized incidence rate 6.6 (95%CI 3.3–13.2)] (184). This may reflect surveillance bias as those patients who go on to develop SLE may have had EBV testing as part of their workup for early SLE symptoms. More recently, there are data to suggest that EBV reactivation is associated with SLE disease onset. In a prospective study of unaffected relatives of SLE patients (n=436), SLE relatives who transitioned to classifiable SLE had increased levels of EBV IgG antibodies prior to SLE transition compared to relatives who did not transition (185). Furthermore, increasing levels of EBV antibodies were associated with SLE disease transitioning, particularly among those with variants in genes that are associated with SLE and implicated in EBV infection.

The association between vaccinations and SLE risk remains to be elucidated, but thus far, epidemiological studies in SLE suggest that there is no association (186). It is thought that vaccines could potentially trigger autoimmunity through molecular mimicry, autoantibodies, and response to adjuvants in the vaccine. There have been emerging reports of new-onset autoimmune diseases including rheumatoid arthritis (187), immune thrombotic thrombocytopenia (188), autoimmune liver disease (189), IgA nephropathy (190), and Guillain-Barré Syndrome [reviewed in (191)] after vaccination. However, the evidence is from mainly case reports or cross-sectional studies demonstrating a temporal association. There have also been a few case reports of SLE and lupus nephritis 1-2 weeks following COVID-19 vaccination (192–194). Without more substantive evidence, however, individuals should be encouraged to get vaccinated as it remains one of the most effective interventions to prevent COVID-19 infection and related morbidity and mortality.

### 2.3 Early or Preclinical SLE

During the next phase of SLE pathogenesis, still pre-diagnosis, individuals may start to develop early non-specific symptoms of SLE, but not yet enough to be diagnosed or classified with the disease (12, 103). These patients are sometimes referred to as incomplete lupus or undifferentiated connective tissue disease (195). Eventually, some people with early and non-specific breakdown of immune tolerance and signs and symptoms of systemic inflammation and autoimmunity will develop more disease features and organ damage and diagnosed or classified as SLE. The duration of this early phase is highly variable from individual to individual. Some may have smoldering disease onset over years, while others experience a rapidly explosive onset of SLE with multiple simultaneous and severe clinical manifestations and autoantibodies. The rapidity of SLE onset likely relates to the specific combination of genetic and environmental SLE risk factors and their interactions, and has been shown to vary by racial ancestry (196). Depending on the cohort and setting, it has been reported that up to half of undifferentiated SARD patients with very early connective tissue disease evolve to fulfill diagnostic and classification criteria of a SARD, including SLE (197). Identifying those at high risk of developing SLE, or in early phases of its development, would enable a “window of opportunity” whereby interventions could be targeted at intercepting disease and halting or slowing the progression to SLE (87).

## 3 Discussion: Proposal of a Clinical Care Pathway to Screen and Prevent SLE

Even before patients are diagnosed with SLE, some may suffer irreversible organ damage, including pulmonary arterial hypertension, cardiovascular disease, renal, and neurological damage (198). Studies have also demonstrated that prior to being diagnosed by an astute clinician or meeting formal classification criteria for SLE, patients are already at higher risk of hospitalizations and lupus-related complications (199, 200). If these patients who are developing SLE could be identified at an early stage, decision‐making regarding preventative strategies and therapeutic interventions could be improved.

**Figure 2:**
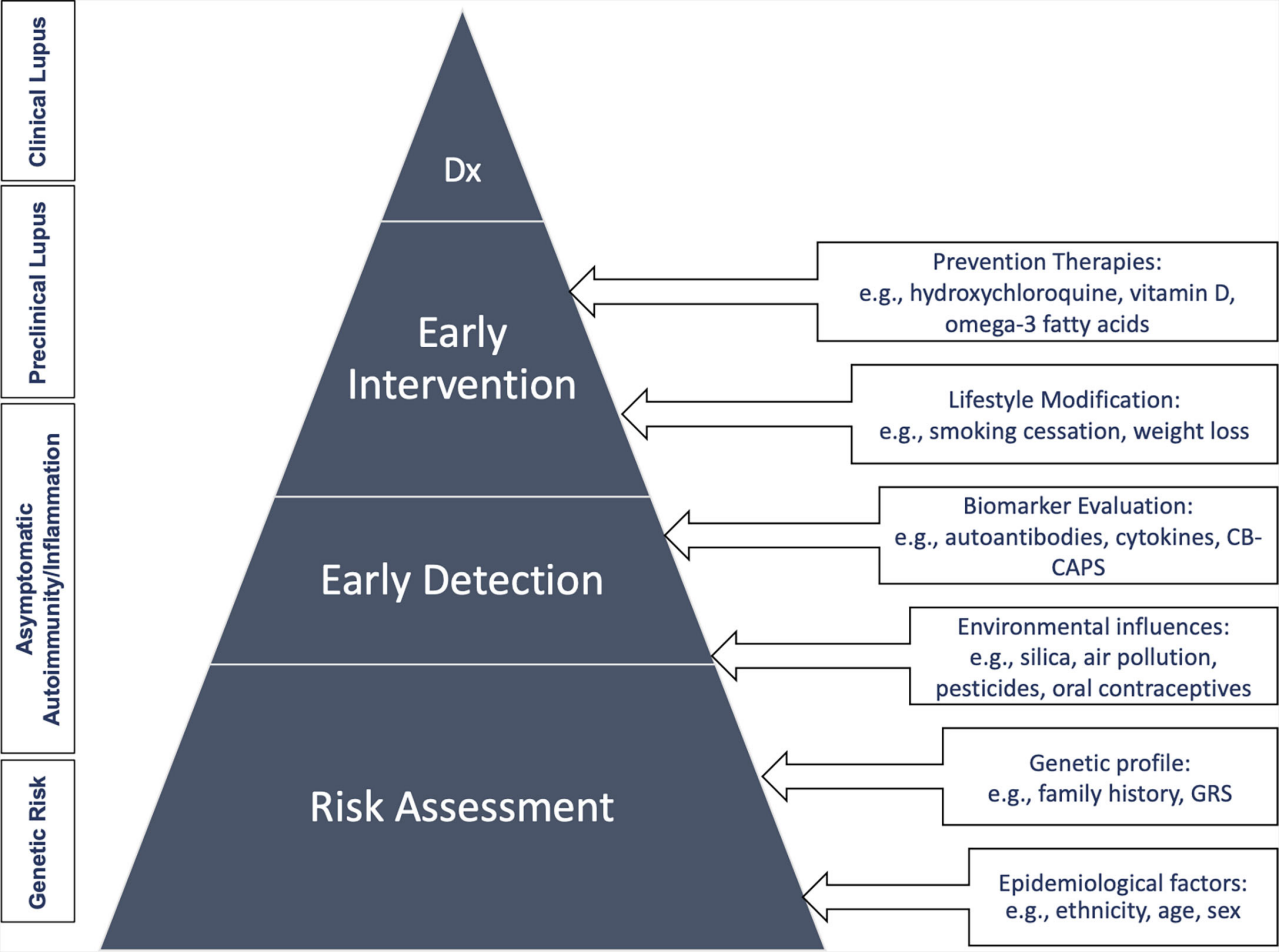
Clinical care pathway for the screening and prevention of SLE. Dx, diagnosis; GRS, genetic risk score.

An appropriate screening and prevention program for SLE has great potential to improve public health outcomes. When organized effectively, it would be targeted to identifying those at risk for SLE to prevent disease development, reduce disability, and cut mortality through early detection and treatment. This will be challenging however, given that SLE is a rare disease in the general population. Here we proposed a clinical care pathway for the screening and prevention of SLE (**Figure 2**) involving four different levels that start with targeting patients who are at genetic risk, the asymptomatic autoimmunity stage, pre-clinical, and finally clinical disease states as discussed in the section above.

### 3.1 Risk Assessment and Early Detection

Currently, there is no consensus concerning how to identify individuals at high risk for SLE or at what preclinical phase of disease should a patient be referred to see a rheumatologist. Given that SLE is a relatively rare disease with an incidence of about 1/2000 in the general population, most hypothetical screening programs would have to rely on inexpensive, readily available, and accurate tests (201). Population studies have used a 30-item questionnaire that can be completed within 30 minutes called the Connective Tissue Disease Screening Questionnaire (CSQ) to screen populations for SLE and other connective tissue diseases (202). It has high sensitivity for SLE (96%, 95%CI 90-99%) but moderate specificity (86%, 95%CI 81-91%) and has been validated among African American women (203). It is best employed in a two-stage screening method followed by medical record review or in-person assessment and should not be used as a test on its own due to the high false-positive rate. The ANA is the biomarker that we utilize today to “screen” for autoimmune connective tissue diseases, including SLE (204–206). However, as the ANA test is most usually performed when patients already have symptoms, it is not really a population-based screening test. As some patients may already have organ damage, ideally, those patients should be caught earlier in the asymptomatic autoimmunity and early preclinical phases, *prior* to clinical signs and symptoms. In this review, we highlighted numerous biomarkers that have shown promise in the identification of at-risk patients that could be detected in these earlier phases (**section 2**). These included autoantibodies such as ANA and anti-SSA/Ro60, genetic susceptibility loci, and upregulated cytokines/chemokines that coincide with timing of the initial appearance of autoantibodies, as well as markers of complement activation.

While some of these tests are readily available and accessible, there are several questions related to their use for screening purposes that need to be clarified. To better understand what makes a screening program appropriate, there are ten principles laid out by the 1968 World Health Organization that prompt important discussion about the benefits, harm, costs and ethics of a screening and prevention programs (207). If a program for SLE were implemented today, it would likely satisfy many of the criteria such as 1) “the condition should be an important health problem”; 2) “there should be an accepted treatment for patients with recognized disease”; 3) “facilities for diagnosis and treatment should be available”. However, there is still uncertainty surrounding some of the other criteria. Specifically related to testing, for instance, it is unclear if a biomarker test or panel were administered to screen for SLE in the general population that, “the cost of case-finding (including a diagnosis and treatment of patients diagnosed) [would] be economically balanced in relation to possible expenditure on medical care as a whole.” We have yet to determine the population that should be targeted for screening. However, it may be reasonable to narrow the screening eligibility criteria, based on the evidence from epidemiological studies to individuals from high-risk populations.

Preliminary data using the NHS and NHSII cohorts demonstrate that a weighted GRS in combination with lifestyle and environmental risk factors predicted future SLE risk with a good area under the curve of 0.77 (208). Therefore, using a GRS in combination with other risk factors assessment may be a valuable tool that may feasibly be employed in at-risk populations for predicting disease (**Table 2**). Once these patients have been identified, they could then be referred and potentially enrolled in prevention trials (discussed in *3.2.2 Preventative Therapies*). Other prevention efforts targeting individuals at high genetic risk for lifestyle modification type of prevention trials could also be envisioned.

**Table 2:** SLE risk stratification chart.

Types of Risk Factors: Epidemiological, immune biomarkers, lifestyle and environmental	Genetic Risk				
	Low Risk -No high-risk alleles -Low GRS -No family history		↔	High Risk -Multiple high-risk alleles -High GRS -Positive family history	
No risk factors	Low Risk	Low Risk	Moderate Risk	High Risk	Very High Risk
1-2 types of risk factors	Low Risk	Low Risk	Moderate Risk	High Risk	Very High Risk
All 3 types of types of risk factors present	Moderate Risk	Moderate Risk	Moderate Risk	High Risk	Very High Risk
All 3 types of types of risk factors present with 1-2 SLE features	High Risk	High Risk	High Risk	High Risk	Very High Risk
3 or more types of risk factors with multiple SLE features but not enough to meet classifiable disease	Very High Risk	Very High Risk	Very High Risk	Very High Risk	Very High Risk

GRS, genetic risk score; SLE, systemic lupus erythematosus.

### 3.2 Early Intervention

#### 3.2.1 Lifestyle Modification

We discussed several modifiable risk factors that health care providers should encourage their patients who may be at risk for SLE to address, including smoking cessation, moderate alcohol consumption, regular exercise, avoidance of certain occupational and environmental exposures, medications, and maintaining a healthy weight and good sleep hygiene. The cost-effectiveness of adopting a healthy lifestyle is clear in that it is not only the risk of SLE that would be reduced, but that of many other chronic and complex diseases. To test the effectiveness of lifestyle interventions in actually reducing SLE risk, a primary prevention clinical trial would be necessary, but would be very challenging.

It is important to recognize that while the evidence suggests providers should encourage patients to adhere to as many healthy behaviors as possible for the greatest reduction in SLE and other chronic disease risk, there are many structural and institutional factors that affect an individual’s ability to adhere or achieve a healthy lifestyle. These include poverty, pollution, toxins, stress, and institutional and structural racism, among others, which have disproportionately affected non-White groups in the United States, who are also the same groups with the highest incidence and severity of SLE. Future studies should examine how to improve adherence to lifestyle interventions and address barriers that prevent or limit ability to meet healthy goals, especially among sociodemographic groups that are medically vulnerable.

#### 3.2.2 Preventative Therapies

The first prevention trial in SLE is the Study of Anti-Malarials in Incomplete Lupus Erythematosus (SMILE), a multi-center, randomized, double-blind, placebo-controlled trial of HCQ compared to placebo, a 24-month clinical study (209). The purpose of this trial is to evaluate the efficacy and safety of HCQ intervention to prevent future onset of clinically apparent SLE. The inclusion criteria are patients 15-49 years of age with a positive ANA and at least one (but not three or more) additional clinical or laboratory criterion from the 2012 SLICC classification criteria (210). This study is expected to be completed in 2023. This study was initiated after James et al. demonstrated in a retrospective study on 130 United States military personnel that individuals who were treated with HCQ prior to SLE diagnosis had delayed the onset of complete SLE compared to untreated patients (median: 1.08 years versus 0.29 years) (18). Furthermore, individuals who had received HCQ in that study had slower accumulation of new autoantibodies. Other small studies showed that patients with incomplete SLE or new-onset, mild SLE treated with HCQ had lower levels of IFN-inducible genes, serum BLyS levels (also known as B cell–activating factor or BAFF), anti-C1q antibodies, IL-9, and better self-reported health status scores (86, 211). These results support the hypothesis that HCQ could influence SLE disease progression. Therefore, the SMILE trial will not only inform clinicians as to whether HCQ can be used to prevent SLE, but it will be the first step towards testing feasibility of disease prevention studies in SLE.

Recently, the results of a large (25,871 participants) randomized, double-blind, placebo-controlled, two-by-two factorial design trial examined the impact of vitamin D (cholecalciferol; 2000 IU/day) and marine omega 3 fatty acids (1 g/day as a fish oil capsule containing 460 mg of eicosapentaenoic acid and 380 mg of docosahexaenoic acid) on the incidence of various autoimmune diseases (212). The investigators found a reduction in autoimmune disease by 22% with vitamin D supplementation for five years, with or without omega 3 fatty acids, reduction by 15% with omega-3 fatty acid supplementation with or without vitamin D (not statistically significant). While there were too few new cases of SLE to be examined in this older population (men age 50 and older and women age 55 and older), vitamin D deficiency is common in SLE (213) and is important for regulating numerous genes involved in inflammation and the immune system through IL-2 inhibition, antibody production, and proliferation of lymphocytes (214, 215). Additionally, prior small cohort studies in SLE on specialized pro-resolving mediators (SPMs), a family of omega-3 fatty acid-derived lipid mediators, suggest that specific SPMs, such as the resolvins and lipoxins, may counter-regulate the production of inflammatory mediators and promote resolution of inflammation (216, 217). Further studies to examine whether omega-3 fatty acid supplementation can affect SPM levels and thereby forestall the development of SLE in at-risk populations will be needed.

Another potential therapy to decrease SLE risk that has been proposed is melatonin. Disrupted melatonin production in nightshift workers has been proposed as an important mechanism of increasing risk for autoimmune diseases including SLE [reviewed in (218)]. In lupus-prone mice, abnormal circadian rhythm of melatonin levels in response to light/dark cycle has been observed (219). When melatonin was administered to lupus-prone mice, there was decreased levels of autoantibodies, inflammatory cytokines, reduce renal injury, and increased levels of anti-inflammatory cytokine IL-10 (220, 221), particularly for females. Further studies in humans are called for to investigate the mechanism by which melatonin may be related to SLE risk and whether it could be a potential therapeutic strategy.

It is important to recognize that there are significant barriers to conducting prevention trials in SLE. A major challenge faced by past SLE prevention trials is low patient recruitment and retention. A lack of enthusiasm among clinicians and patients due to risk aversiveness and misunderstanding or misinterpretation of the purpose of prevention trials have resulted in underenrollment and selective enrollment, poor adherence, and attrition in some studies (222–224). Whereas good health status, encouragement from one’s physicians, desire to learn and contribute to research are positive factors for participation in SLE prevention trials (225). Therefore, future prevention trials in SLE should employ strategies such as health education about the clinical problem and importance of the trial, and involving the patients personal physicians to improve recruitment of SLE patients into prevention trials (225).

## 4 Conclusion

Developing a deeper understanding of SLE pathogenesis, its preclinical stages, and risk factors, will ultimately enable effective screening and potentially prevention. This may appear to be a daunting task; however, tremendous progress has been made over the last few decades with greater insights into the etiopathogenesis of SLE, identification of novel biomarkers for early SLE detection, epidemiologic and genetic studies that have revealed important risk factors, and the first prevention trial in SLE is already underway. Well-designed prospective clinical studies to further elucidate the mechanisms of disease development and more clinical prevention trials are needed.

## Author Contributions

All authors have participated drafting the work or revising it critically for important intellectual content, final approval of the version published, agreement to be accountable for all aspects of the work in ensuring that questions related to the accuracy or integrity of any part of the work are appropriately investigated and resolved,

## Funding

This work was supported by the Lupus Foundation of America Gary S. Gilkeson Career Development Award and NIH K24 AR066109 and R01 AR057327, and McCaig Institute for Bone and Joint Health.

## Conflict of Interest

The authors declare that the research was conducted in the absence of any commercial or financial relationships that could be construed as a potential conflict of interest.

## Publisher’s Note

All claims expressed in this article are solely those of the authors and do not necessarily represent those of their affiliated organizations, or those of the publisher, the editors and the reviewers. Any product that may be evaluated in this article, or claim that may be made by its manufacturer, is not guaranteed or endorsed by the publisher.
